# Supplementary material for: Identification of photoperiod-induced specific miRNAs in the adrenal glands of Sunite sheep (Ovis aries)
Source: Front Vet Sci. 2022 Jul 22;9:888207. doi: 10.3389/fvets.2022.888207 (PMC9354845; doi:10.3389/fvets.2022.888207)
Supplement: Supplementary file 1 [file Data_Sheet_1.ZIP › Supplementary file/miRDeep2 file.zip!/miRDeep2 file/Know_Structure/SP42A3/oar-mir-299.pdf]

miRBase precursor : oar-mir-299
Total read count : 140
oar-miR-299-5p read count : 140
oar-miR-299-3p read count : 0
remaining reads : 0

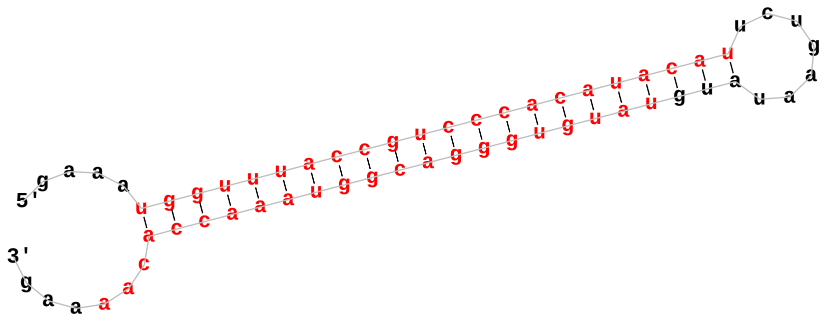

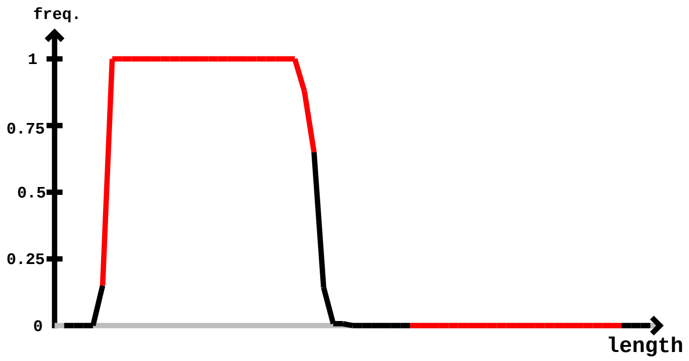


```
         oar-miR-299-5p
                                            oar-miR-299-3p
5'- gaaauggguuuaccgucccacauacauucugaauaugauaugugggacgguaaaccacaaaag   -3'    exp
    ....((((((((((((((((((((((.......))))))))))))))))))))))......     reads      mm         sample
    ...augguuuaccgucccacauac...................................     5          0          seq
    ...augguuuaccgucccacauaca..................................     16         0          seq
    ....ugguuuaccgucccacauac...................................     12         0          seq
    ....ugguuuaccgucccacauaca..................................     16         0          seq
    ....ugguuuaccgucccacauacau.................................     71         0          seq
    ....ugguuuaccgucccacauacauu................................     13         0          seq
    ....ugguuuaccgucccacauacauA................................     6          1          seq
    ....ugguuuaccgucccacauacauucu..............................     1          0          seq
```
